# Pelvic floor disorders associated with higher-level sexual dysfunction in the Kersa district, Ethiopia

**DOI:** 10.4274/tjod.86658

**Published:** 2019-01-09

**Authors:** Merga Dheresa, Alemayehu Worku, Lemessa Oljira, Bezatu Mengistie, Nega Assefa, Yemane Berhane

**Affiliations:** 1 Haramaya University College of Health and Medical Sciences, School of Nursing and Midwifery, Harar, Ethiopia; 2 Addis Ababa University College of Health and Medical Sciences, Faculty of Public Health, Addis Ababa, Ethiopia; 3 Haramaya University College of Health and Medical Sciences, School of Public Health, Harar, Ethiopia; 4 Haramaya University College of Health and Medical Sciences, School of Public Health, Department of Environmental Health Science, Harar, Ethiopia; 5 Addis Continental Institutes of Public Health, Addis Ababa, Ethiopia

**Keywords:** Sexual dysfunction, physiological, pelvic floor diseases, women, Ethiopia

## Abstract

**Objective:**

To assess the prevalence of female sexual dysfunction and its association with pelvic floor disorder (PFD) in a large scale, community-based study.

**Materials and Methods:**

A total of 2389 women who were married and still in union at the time of the study were drawn from 3432 women who had ever been married who participated in a PFD study. Study participants were selected through a multistage sampling procedure based on Kersa Health and Demographic Surveillance System database. The Female Sexual Function Index questionnaire was employed to collect data. The index score <26.55 was used as a cut-off point for sexual dysfunction. The content of the tool was validated and internal reliability was checked using Cronbach’s alpha. Poisson regression model with robust variance estimation was used to investigate the relationship between PFDs and sexual dysfunction.

**Results:**

From the total 2389 participants, 1127 [47.0%; 95% confidence interval (CI): 45.0-49.0] had sexual dysfunction. Sexual desire disorder was the most prevalent disorder (72.0%; 95% CI: 70.0-74.0). After controlling for confounding factors, the prevalence of female sexual dysfunction was found as 56% (adjusted prevalence ratio, 1.56; 95% CI: 1.44-1.69) higher with women with PFD as compared with women without PFD.

**Conclusion:**

In the rural community of Kersa, about half of the women have sexual dysfunction and it is significantly associated with PFD. This would call for an urgent intervention against PFD to maximize the women’s sexual and reproductive health.

**PRECIS:** Using Female Sexual Function Index questionnaire, 47% of participants were identified having sexual dysfunction, and sexual dysfunction was 56% higher with women with pelvic floor disorder as compared to women without pelvic floor disorder.

## Introduction

Sexual dysfunction is a heterogeneous group of disorders characterized by a clinically significant disturbance in a person’s ability to respond sexually or to experience sexual pleasure^(1)^. Sexual dysfunction comprises physical, social, and psychological dimensions of disturbance. It can affect any phase of sexual functioning including desire, arousal, orgasm, satisfaction, lubrication, and pain^(2)^. Sexual dysfunction has detrimental impacts on women’s quality of life, mainly on their interpersonal relationships, ability of reproduction, and psychological well-being^(3,4)^.

Globally, 41% of women have sexual dysfunction. This problem is more prevalent among African women (62%)^(5)^. Viewing sex as a method of procreation (in contrast to pleasure), genital mutilation, gender inequality, and poor reproductive health conditions fuel the burden of female sexual dysfunction (FSD) in developing regions^(5,6,7)^.

Pelvic floor disorders (PFDs), whose symptoms involve urinary incontinence, over-active bladder (OAB), pelvic organ prolapse (POP), and fecal incontinence, can adversely affect the sexual function and satisfaction of women^(8)^. Women with PFD fail to attain sexual function due to discomfort, mechanical obstruction of prolapsed organ, pain, and leakage. For these reasons and due to the fear of incontinence^(9)^, they avoid sexual intercourse or restrict sexual activity. In addition to its physical effect, PFD causes women to develop low self-esteem, negative self-image especially about their body, and depression. It is generally believed that all these directly affect the women’s relationship with their partner and aggravate in them sexual dysfunctions^(10,11)^. Yet, the available research findings on the relationship between PFD and sexual function remains indeterminate, and, in many cases, contradicting. Some studies report that sexual dysfunction makes little or no difference between women with and without PFD^(12,13)^. Whereas in other studies, PFD relates to FSD in bivariate analysis, but loses its relation when adjusted for other variables^(13,14)^. Furthermore, other studies quite intriguingly show that PFD is significantly associated with sexual dysfunction^(4,15)^.

Added to the aforementioned contradictory and indeterminate stories, in developing countries such as Ethiopia, sexual behaviors, activities, and problems are not discussed openly. To discuss these is considered an utter social taboo when it comes to women, particularly in the Ethiopian context. Partly due to this, the prevalence and burden of FSD remains largely less studied or unknown. Therefore, this study attempts to assess the prevalence of sexual dysfunction as well as examine the relationship between PFD and sexual dysfunction among women with and without PFD in Eastern Ethiopia.

## Materials and Methods

This study is part of a larger community-based cross-sectional study, which was established to investigate factors associated with PFDs. The study selected the Kersa Health and Demography Surveillance System (HDSS), Kersa District, Ethiopia, as its setting. The study was conducted from August 10th, to September 4^th^, 2016.

From among 3432 women who had ever been married participating in the PFD study, 2389 women who were married and still in union at the time of the study were selected. At the time of study, they had been residing inside Kersa HDSS for at least six months. A multi-stage, stratified, random sampling procedure proportional to the size of the household in each kebeles (small administrative unit in Ethiopia) was used to enroll the study participants. The Kersa HDSS database was used as a sampling frame. The study was approved by Haramaya University Health and Medical Sciences College Institutional Health Research ethics review committee (approval number: IHRERC/001/2016). Consent form was filled out by all participants.

### Data collection tools

A standardized Female Sexual Function Index (FSFI) questionnaire was adopted and distributed in order to measure sexual function^(2,16)^. A PFD questionnaire was customized and adapted from the literature^(17)^. The socio-demographic and obstetric conditions of the participants were also collected using a structured questionnaire adopted form a national survey document^(18)^. The contents of the questionnaires were validated by three gynecologists and two reproductive health experts. Further, the questionnaires were pre-tested in a similar setting and refined. This was done to enhance the appropriateness and fitness of the tools to the social and cultural norms. Experienced female data collectors and field supervisors fluent in the local languages were recruited and trained for four days before the fieldwork. The training included field procedures, interviewing techniques, and discussion on the content of the tools. Field supervisors checked compliance to field procedures and the completeness of the questionnaires in the field. The data collectors undertook interviews with the participants in private settings in the interviewees’ homes.

The study protocol was approved by Haramaya University Health and Medical Sciences College Institutional Health Research Ethics Review Committee. Written informed consent was obtained from each participant. In order to protect the confidentiality of the information, names and identification were not included in the written questionnaires.

### Measurement

Sexual function, the outcome variable for this study, was evaluated using the FSFI questionnaire, which is a 19-item inventory measuring sexual function over a 4-week period in six domains. The domains include desire (items 1 and 2), arousal (items 3-6), lubrication (items 7-10), orgasm (items 11-13), satisfaction (items 14-16), and pain (items 17-19). The response format of four of the items is a 5-point Likert scale. Other items are scored on a scale from 0 to 5, with zero score representing “no sexual activity”. Ratings of 1 to 5 indicate level of sexual functioning (e.g., 1: extremely difficult, 2: very difficult, 3: difficult, 4: slightly difficult, 5: not difficult)^(16)^. By adding the scores of the individual items that comprise the domain and multiplying the sum by domain factor, individual domain scores were obtained. Then, the total sexual function score was obtained by adding the six domain scores. The full-scale score range is from 2.0 to 36.0 with higher scores indicating better function. Women with total FSFI scores less than 26.55 were classified as having sexual dysfunction^(2,16)^. Sexual function of each domain was categorized based on a cut-off point provided in the literature^(2)^. Thus, the outcome variable was dichotomized into having sexual dysfunction or not. We checked the internal consistency of the FSFI in this study setting; the FSFI scale had good psychometric properties, with high internal consistency (Cronbach’s alpha values between 0.85 and 0.94). The independent variables includd PFDs, obstetrics history, and socio-demographic characteristics. PFD was assessed using an interviewer-administered questionnaire. The questionnaire was customized and adapted from the literature^(17)^. Each PFD [stress urinary incontinence (SUI), OAB, POP, and anal incontinence] was dichotomized as present or absent based on the responses to each symptom domain. PFDs were dichotomized as women with or without PFD. The detail of this section has been described in detail elsewhere^(19)^. Obstetrics history was coded as follows: parity was coded in para 1-4 and para 5 and above; history of abortion was coded as “yes” or “no”; age at first child birth was categorized into less than 18 and 18-and-above years; age at first marriage was grouped into 10-14, 15-19, and 20-and-above years.

### Statistical Analysis

The obtained data were double-entered into Epi-Data 3.1 and validated using the same statistical software. Then, the data were analyzed using STATA version 14. The overall prevalence of sexual dysfunction with in all domains was obtained with 95% confidence intervals (CI). The proportion of sexual dysfunction among women with PFD was also obtained with 95% CI. When the outcome of interest was common (more than 10%), the odds ratio overestimated the prevalence ratio (PR) and logistic regression model produced poor estimates^(20)^. Hence, the Poisson regression analysis model was employed with robust variance estimation in order to investigate the relationship between PFD and sexual function. Bivariate analysis was first made and the variables with a p value less than 0.2 were included to the subsequent model building. Model 1 was built to examine the association of socio-demographic and personal behaviors with FSD. Subsequently, relevant obstetrics history variables were included into model 2 to assess their relationship with FSD when controlling for socio-demographic and personal behaviors. The final model (model 3) was built using PFD variables and all variables in model 1 and model 2 were employed to assess the relationship between PFD and FSD. These were reported in an adjusted PR (APR) with 95% CI. Multi-collinearity was assessed using variance inflation factors. Interaction was also checked among the independent variables.

## Results

**Figure 1:**
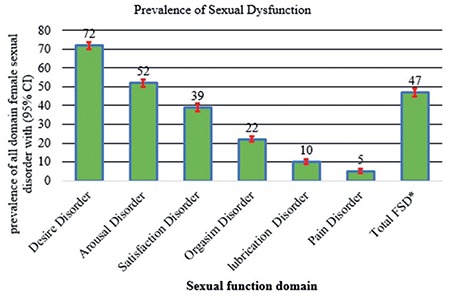
Prevalence of female sexual dysfunction among women living in Kersa Health and Demography Surveillance System, Ethiopia, 2016 *FSD: Female sexual dysfunction, CI: Confidence interval

**Figure 2:**
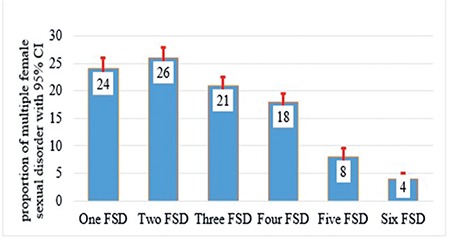
Prevalence of multiple sexual dysfunction among women living in Kersa Health and Demography Surveillance System, Ethiopia, 2016 FSD: Female sexual dysfunction, CI: Confidence interval

**Figure 3:**
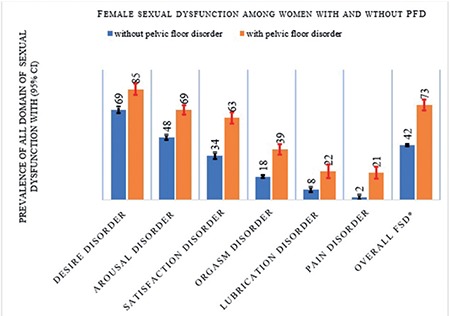
Prevalence of sexual dysfunction among women with and without pelvic floor disorder living in Kersa Health and Demography Surveillance System, Ethiopia, 2016 FSD: Female sexual dysfunction, CI: Confidence interval, PFD: Pelvic floor disorder

The mean age [± standard deviation] of the participants was 32±10 years. The mean gravidity was 5±3 and the mean parity was 5±2. The majority (74%) of the participants did not attend school and 65% reported that they consumed Khat (*Catha edulis*), a stimulant leaf commonly consumed in the study area. Ten percent of the participants had married more than once. Eighteen percent had at least one type of PFD (Table 1). Forty-seven percent (95% CI: 45-49) of the respondents had sexual dysfunction. Sexual desire disorder was the most prevalent form of FSD (72.0%; 95% CI: 70-74), followed by arousal disorder (52%; 95% CI: 50-54), and pain disorder, the least being 5.0% (95% CI: 4-6) (Figure 1). Among women with sexual dysfunction, 24.0% had only one type of sexual dysfunction, 26.0% had two types, and 4.0% had all six types of sexual dysfunction (Figure 2). The prevalence of desire disorder was 85.0% (95% CI: 81-88) among women with PFD, whereas it was 69.0% (95% CI: 67-71) among women without PFD (Figure 3). Women with PFD were more likely to report sexual dysfunction than women without PFD. Adjusting for other characteristics did not affect the estimation of the association between PFD and sexual dysfunction. Finally, a Poisson regression model was used to adjust for socio-demographic data, personal behaviours, and obstetric variables. Nevertheless, the result showed that PFD maintained its association (APR, 1.56; 95% CI: 1.44-1.69). Moreover, the results indicated that there was a significant relationship between educational level and sexual dysfunction. That is, sexual dysfunction increased by 31% (APR, 1.31; 95% CI: 1.15-1.50) among uneducated women compared with educated women. Also, the result indicated that consuming Khat and grand multi-parity were associated with a 12% and 41% increase in the prevalence of sexual dysfunction (APR, 1.12; 95% CI: 1.02-1.24) and (APR, 1.41; 95% CI: 1.28-1.56), respectively (Table 2).

## Discussion

As was illustrated above, 47.0% of the participants in this study had sexual dysfunction. About three-quarters of them had sexual desire disorder, and 1 in 20 had pain disorder. PFD was found to be an independent associated factor for FSD. Our results documented that sexual dysfunction was more prevalent among women with PFDs. In other words, the findings indicate that PFD affects women’s reproductive and sexual health. The 47% prevalence of sexual dysfunction in this study is consistent with studies conducted in different parts of the world^(5,21,22)^. This high prevalence of sexual dysfunction can causes poor quality of life, relationship breakdown and unhappiness that leads to divorce if the couple are unable to resolve it^(8,22,23)^. Biopsychosocial factors like female genital mutilation, poor interpersonal relationship with spouse, lack of privacy and uncomfortable surrounding, and cultural taboos that prevent open discussion on sexual issues exacerbate negative sexual functions^(5,7,24)^. Among the six domains of sexual function, desire disorder (72.0%), and arousal disorder (52.0%) are the most frequently reported symptoms in this study. Pain disorder is the least reported problem with 5%. Desire and arousal are co-occurring event in sexual process and they share a common latent factors^(25)^. Our finding is consistent with other study’s finding^(26)^. This high prevalence of sexual desire and arousal disorder in this study might be explained by the generic idea that women in traditional societies should not show sexual desire for it is a taboo for women to express or show her sexual desire^(6,7,25)^. In this study, 39.0% and 22.0% of the women had satisfaction and orgasmic disorders, respectively. A possible explanation for this is that these women experience limited sexual education, lack of awareness on genital anatomy and function, and poor sexual relationship. It is worth nothing, that satisfaction disorder had a direct link to marital and partner relationship quality^(21)^. Moreover, in this study, the prevalence of FSD increased by 56.0% among women with PFD compared to women without PFD, a finding which is consistent with other studies^(4,27,28)^. Pelvic floor function has a direct role in maintaining genital arousal and orgasm. Since weak contraction of pelvic floor muscles reduce sexual function, PFD has a great impact on sexual function^(29)^. The anatomical and physiological problem in pelvic floor in relation to POP and SUI interferes with sexual function because women lose sexual desire, unable to attain orgasm, and feel pain during intercourse. All these are, in turn, caused by the underlying problem of PFD and the women’s negative self-image of unattractiveness in relation to changes to their bodies. In addition to this, women with PFD exhibit poor quality of life and low self-esteem both of which further exacerbate sexual dysfunction^(9,11)^. Parity is significantly associated with FSD. Large number of children can negatively affect the intimacy of couples, and lower marital sexual relationships^(4,30)^. Educated women have increased awareness of sexual needs and rights and are more likely to have positive sexual experience^(5,30)^. 

Sexual dysfunction was 12% higher among Khat (*Catha edulis*) consumed participants compared to non Khat (*Catha edulis*) consumer participants. Khat is a psychostimulant plant commonly chewed in certain countries of East Africa^(31)^.The relationships of Khat (*Catha edulis*) chewing and sexual function is not clear. Studies revealed that consuming Khat (*Catha edulis*) has a negative impact on male sexual function by inducing erection problem and impotence. Its relationship with female sexual function is not yet established^(31,32)^. In general, this study was conducted in an established Health and Demographic Surveillance System, which provided a robust platform to randomly select participants, thus it is possible to generalize this finding to women in rural Eastern Ethiopia. In addition, we used the FSFI questionnaire, which has been tested in different parts of the world; the questionnaire has good internal consistency.

### Study Limitations

The findings relied on self-reported data, which are subject to recall and desirability bias. We would like to stress that, because revealing sexual function is associated with cultural taboos, the participant women in this study might have hesitated to adequately respond to the data collectors. Thus, they might have underreported their sexual experiences. Consequently, this might underestimate the prevalence of sexual dysfunction. To minimize the impact of these aspects, the participants were interviewed by female data collectors in a private setting.

## Conclusion

About half of the women in the study community had sexual dysfunction. PFDs increase the prevalence of sexual dysfunction by 56%. This calls for an urgent need to initiate interventions against PFD to promote womens’ reproductive health.

## Figures and Tables

**Table 1:**
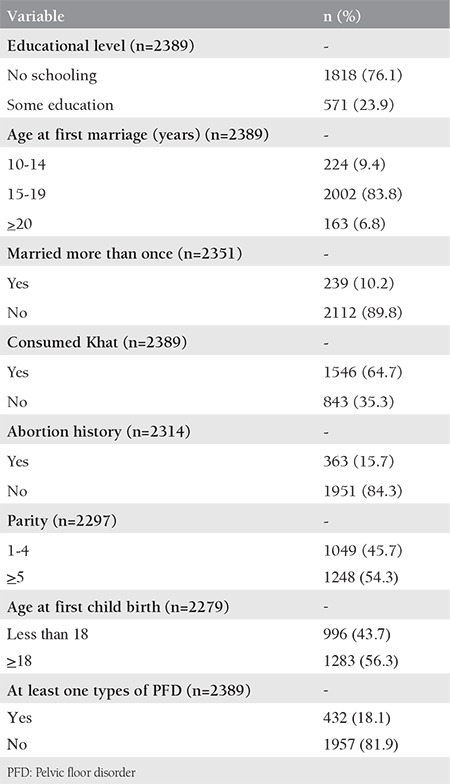
Socio-demographic characteristics and reproductive health history among women in sexual relationships living in Kersa Health and Demography Surveillance System, Ethiopia 2016

**Table 2:**
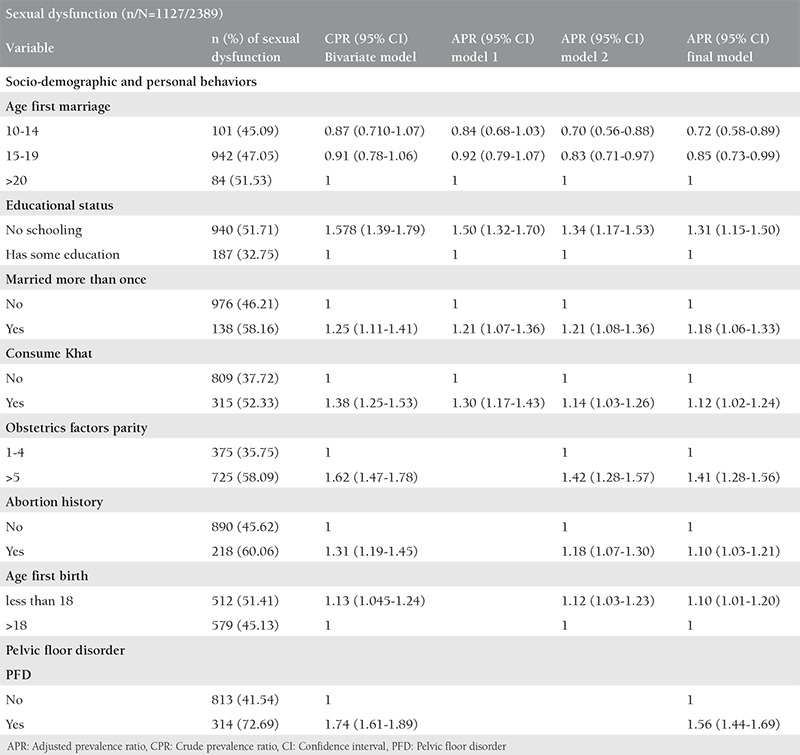
Factors associated with female sexual dysfunction among ever married women living in Kersa Health and Demography Surveillance System, Ethiopia 2016
